# An Improved Multimodal Medical Image Fusion Approach Using Intuitionistic Fuzzy Set and Intuitionistic Fuzzy Cross-Correlation

**DOI:** 10.3390/diagnostics13142330

**Published:** 2023-07-10

**Authors:** Maruturi Haribabu, Velmathi Guruviah

**Affiliations:** School of Electronics Engineering, Vellore Institute of Technology, Chennai 600127, India

**Keywords:** medical imaging, image fusion, disease diagnosis, intuitionistic fuzzy set, intuitionistic fuzzy image, subjective and objective analysis

## Abstract

Multimodal medical image fusion (MMIF) is the process of merging different modalities of medical images into a single output image (fused image) with a significant quantity of information to improve clinical applicability. It enables a better diagnosis and makes the diagnostic process easier. In medical image fusion (MIF), an intuitionistic fuzzy set (IFS) plays a role in enhancing the quality of the image, which is useful for medical diagnosis. In this article, a new approach to intuitionistic fuzzy set-based MMIF has been proposed. Initially, the input medical images are fuzzified and then create intuitionistic fuzzy images (IFIs). Intuitionistic fuzzy entropy plays a major role in calculating the optimal value for three degrees, namely, membership, non-membership, and hesitation. After that, the IFIs are decomposed into small blocks and then perform the fusion rule. Finally, the enhanced fused image can be obtained by the defuzzification process. The proposed method is tested on various medical image datasets in terms of subjective and objective analysis. The proposed algorithm provides a better-quality fused image and is superior to other existing methods such as PCA, DWTPCA, contourlet transform (CONT), DWT with fuzzy logic, Sugeno’s intuitionistic fuzzy set, Chaira’s intuitionistic fuzzy set, and PC-NSCT. The assessment of the fused image is evaluated with various performance metrics such as average pixel intensity (API), standard deviation (SD), average gradient (AG), spatial frequency (SF), modified spatial frequency (MSF), cross-correlation (CC), mutual information (MI), and fusion symmetry (FS).

## 1. Introduction

In past decades, image fusion has matured significantly in the application fields such as medical [1], military [2,3], and remote sensing [4]. Image fusion is a prominent application in the medical field for better analysis of human organs and tissues. In general, the medical image data is available from various imaging techniques such as magnetic resonance imaging (MRI), magnetic resonance angiography (MRA), computed tomography (CT), T1-weighted MR, T2-weighted MR, positron emission tomography (PET), and single-photon emission computed tomography (SPECT) [5]. Each technique has different characteristics.

Multimodal medical images are widely characterized into two types: anatomical and functional modalities, respectively. Anatomical modalities are, namely, MRI, MRA, T1-weighted MR, T2-weighted MR, and CT. CT images represent a clear bone structure with lower distortion but do not distinguish physical changes, while MRI images provide delicate tissue information with high spatial resolution. CT imaging is used to diagnose diseases such as muscle disease, vascular conditions, bone fractures and tumors etc. MRI imaging is used to diagnose various issues in medial regions such as brain tumors, multiple sclerosis, lung cancer and treatment, brain hemorrhage, and dementia etc. Magnetic resonance angiography, or MRA, is a subset of MRI that utilizes magnetic fields and radio waves, which create images of the body’s arteries, helping clinicians to detect blood flow abnormalities. The weighted MR-T1 images reveal fat, while weighted MR-T2 images provide water content.

Functional modalities are PET, and SPECT. PET imaging gives functionality of human organs with high sensitivity. The PET imaging technology is used to diagnosis different diseases such as Alzheimer’s disease, Parkinson’s disease, cerebrovascular accident, and hematoma. The other application areas of PET imaging are lung and breast cancer diagnosis, and cancer treatment.

SPECT imaging provides blood flow information with minimal spatial resolution, and is used for different diagnoses, namely, brain and bone disorders, and heart problems. The application areas in SPECT imaging are pelvis irradiation detection and treatment, vulvar cancer, breast cancer assessment, and head and neck cancer diagnosis [6,7]. However, single medical image data cannot provide the required information for diagnosis. To overcome this, multimodal medical image fusion is necessary.

Multimodal medical image fusion is the process of merging different modalities of medical images into a single output image. Its advantages include decreased uncertainty, resilient system performance, and higher reliability, all of which contribute to more accurate diagnosis, thus improving treatment. From the literature, authors have reported various multimodality combinations. Fusion of T1- and T2-weighted MR images produce a fused image, and is used to identify tumor regions [8]. The soft and hard tissue information from MRI and CT images, respectively, are combined into a single resultant image by fusion resulting in better image analysis [9]. The T1-weighted MR and MRA [10] combination provides perfect lesion locations with delicate tissues. The MRI–PET [11] combination and MRI–SPECT [12] combinations provide anatomical and functional information in a single image, which is used to better diagnosis disease and medical-related problems. The objective of this research article is to examine the relevance and advancement of information fusion approaches in medical imaging for investigation of clinical aspects and better treatment.

In any fusion strategy, two important requirements should be satisfied: it should not add any artifacts or blocking effects to the resultant image; and no information should be lost throughout the fusion process.

Image fusion techniques are broadly classified into three levels [13], namely, pixel-level, feature-level, and decision-level. In pixel-level fusion, image pixel values are directly merged. In feature level fusion, various salient features are involved in the fusion process such as texture and shape. In decision-level fusion, the input images are fused based on multiple algorithms with decision rules.

## 2. Related Works

The preeminent research issue in medical image processing is to obtain maximum content of information by combining various modalities of medical images. Various existing techniques are included in this literature such as the simple average (Avg), maximum, and minimum methods. The average method provides a fused image with low contrast, while the maximum and minimum methods provide the less enhanced fused images. The Brovey method [14] gives color distortions. Hybrid fusion methods such as the intensity-hue saturation (IHS) and principal component analysis (PCA) [15] combination provides a degraded fused image with spatial distortions. However, the pyramid decomposition-based method [16] shows better spectral information, but the required edge information is not sufficient. Discrete cosine transform (DCT) [17] and singular value decomposition (SVD) [18] methods give a fused image, which has a more complementary nature but does not show clear boundaries of the tumor region. The multi-resolution techniques, such as discrete wavelet transform (DWT) [19], provides better localization in time and frequency domains, but cannot give the shift-invariance due to down-sampling. To overcome this the redundant wavelet transform (RWT) [20] was employed. However, the above technique is highly complex and cannot provide sufficient edge information. The contourlet transform (CONT) technique [21] provides more edge information in a fused image but does not provide the shift invariance. Shift invariance is the most desirable property and is applied in various applications of image processing. These are: image watermarking [22], image enhancement [23], image fusion [24], and image deblurring [25]. The above mentioned drawbacks are addressed by the non-subsampled contourlet transform (NSCT) [26] and non-subsampled Shearlet transform (NSST) [27,28]. Hybrid combinations of fusion techniques such as DWT and fuzzy logic [29] provide a fused image with low contrast because of the higher uncertainties and vagueness, which is present in a fused image.

In general, medical images have poor illumination which means low contrast and poor visibility in some parts, which indicates uncertainties and vagueness. Visibility and enhancement are the required criteria in the medical field to diagnose the disease accurately. In the literature, various image enhancement techniques are reported, namely, gray-level transformation [30] and histogram-based methods [31]. Yet, these methods are not properly improving the quality of medical images. Zadeh [32] proposed a mathematical approach, namely, a fuzzy set in 1965. This fuzzy set approach has played a significant role by removing the vagueness present in the image. However, it did not eliminate the uncertainties. A fuzzy set does not provide reasonable results regarding more uncertainties because it considers only one uncertainty. This uncertainty is in the form of membership function, that lies between the range 0 to 1, where zero indicates the false membership function, and one indicates the true membership function. In the year 1986 Atanassov [33] proposed a generalized version of the fuzzy set i.e., intuitionistic fuzzy set (IFS), which handles more uncertainties in the form of three degrees. These degrees are membership, non-membership, and hesitation degrees. The IFS technique is highly precise, and flexible in order to handle uncertainties and ambiguity problems. 

In this literature review, the research gaps and drawbacks of various medical image fusion techniques are discussed and listed in Table 1:

The main contribution of this research article is described as follows:

- A novel intuitionistic fuzzy set is used for the fusion process, which can enhance the fused image quality and complete the fusion process successfully.
- The intuitionistic fuzzy images are created by using the optimum value, *α*, which can be obtained from intuitionistic fuzzy entropy.
- The Intuitionistic cross-correlation function is employed to measure the correlation between intuitionistic fuzzy images and then produce a fused image without uncertainty and vagueness.
- The proposed fusion algorithm proves that the fused image has good contrast and enhanced edges and is superior to other existing methods both visually and quantitatively.

## 3. Materials and Methods

Intuitionistic fuzzy set (IFS) is used to solve the image processing tasks with membership and non-membership functions [34]. The implementation of IFS is briefly explained, starting from a fuzzy set. 

Let us consider, a finite set *P* is

(1) $$P=\left\{p_{1},p_{2},p_{3},\ldots \ldots \ldots ,p_{n}\right\}$$

A fuzzy set *F* in a finite set *P* is numerically represented as:

(2) $$F=\left\{\left(p,\mu_{F}\left(p\right)\right)\left|p\in P\right.\right\}$$

where $\mu_{F}\left(p\right)$ indicates the membership function of *p* in *P*, which lies between [0–1], and the non-membership function can be represented as $v_{F}\left(p\right)$ and will be equal to $1-\mu_{F}\left(p\right)$. The IFS was introduced by Atanassov [33] in 1986, which considers both $\mu_{F}\left(p\right)\;\mathrm{and}\;v_{F}\left(p\right)$ functions, holding $\mu_{F}\left(p\right)\to \left[0,1\right]\;\mathrm{and}\;v_{F}\left(p\right)\to \left[0,1\right]$. The representation of intuitionistic fuzzy set (IFS) *F* in *P* in a mathematical form, is written as:

(3) $$F=\left\{\left(p,\mu_{F}\left(p\right),v_{F}\left(p\right)\right)\left|p\in P\right.\right\}$$

Which holds the condition $0\leq \mu_{F}\left(p\right)+v_{F}\left(p\right)\leq 1$. However, due to lack of knowledge while characterizing the membership degree, a novel parameter was introduced, called hesitation degree $\pi_{F}\left(p\right)$, by Szmidt and Kacpryzyk [35], for each element *p* in *F*. This can be written as:

(4) $$\pi_{F}\left(p\right)=1-\mu_{F}\left(p\right)-v_{F}\left(p\right)$$

where $0\leq \pi_{F}\left(p\right)\leq 1$.

Finally, based on the hesitation function, the IFS can be represented as

(5) $$F=\left\{\left(p,\mu_{F}\left(p\right),v_{F}\left(p\right),\pi_{F}\left(p\right)\right)\left|p\in P\right.\right\}$$

This article proposed a new intuitionistic fuzzy set-based medical image fusion that is superior for better diagnosis. Initially, the input images are fuzzified and then create intuitionistic fuzzy images with the help of the optimal value, *α*, which can be generated by intuitionistic fuzzy entropy (IFE) [36]. After that, the two intuitionistic fuzzy images are split into several blocks and then apply the intuitionistic fuzzy cross-correlation fusion rule [37]. Finally, the enhanced fused image can be obtained without uncertainty by rearrangement of blocks and accompanied by a defuzzification process. 

### 3.1. Intuitionistic Fuzzy Generator

A function $\phi \left(p\right):\left[0,1\right]$ is called an intuitionistic fuzzy generator (IFG) [38] if $\phi \left(p\right)\leq 1-p,\forall p\in \left[0,1\right]$ and $\phi \left(0\right)\leq 1$, $\phi \left(1\right)\leq 0$, which is a decreasing, continuous, and increasing function, and these are used for the construction of IFS. The fuzzy complements are calculated from the complement function, which is described as:

(6) $$N\left(\mu_{F}\left(p\right)\right)=g^{-1}\left(g\left(1\right)-g\left(\mu_{F}\left(p\right)\right)\right)$$

where g(.) is an increasing function with $g\left(0\right)=0.$ Some of the authors suggested different intuitionistic fuzzy generators using an increasing function, such as Sugeno’s [39], Roy Chowdhury and Wang [40]. 

### 3.2. Proposed Fuzzy Complement and Intuitionistic Fuzzy Generator

In this article, a novel fuzzy complement is created using an increasing function, which is described as:

(7) $$g\left(\mu_{F}\left(p\right)\right)=\frac{1}{\alpha }\log\left(1+\alpha \left(1+e^{-\alpha }\right)\mu_{F}\left(p\right)\right)$$

With $g\left(0\right)=\frac{1}{\alpha }\log\left(1\right)=0$, and $g\left(1\right)=\frac{1}{\alpha }\log\left(1+\alpha \left(1+e^{-\alpha }\right)\right)$.

With the inverse function of $g\left(\mu_{F}\left(p\right)\right)$ is

(8) $$g^{-1}\left(\mu_{F}\left(p\right)\right)=\frac{e^{\alpha \mu_{F}\left(p\right)}-1}{\alpha \left(1+e^{-\alpha }\right)}$$

Substituting the value of $g\left(\mu_{F}\left(p\right)\right)$ in Equation (6), we get

(9) $$N\left(\mu_{F}\left(p\right)\right)=g^{-1}\left(\frac{1}{\alpha }\log\left(1+\alpha \left(1+e^{-\alpha }\right)\times 1\right)-\frac{1}{\alpha }\log\left(1+\alpha \left(1+e^{-\alpha }\right)\mu_{F}\left(p\right)\right)\right)\;N\left(\mu_{F}\left(p\right)\right)=g^{-1}\left(\frac{1}{\alpha }\log\left(\frac{1+\alpha \left(1+e^{-\alpha }\right)}{1+\alpha \left(1+e^{-\alpha }\right)\mu_{F}\left(p\right)}\right)\right)$$

By the induction method, the Equation (8) becomes

(10) $$g^{-1}\left(\frac{1}{\alpha }\log\left(\frac{1+\alpha \left(1+e^{-\alpha }\right)}{1+\alpha \left(1+e^{-\alpha }\right)\mu_{F}\left(p\right)}\right)\right)=\frac{e^{\alpha \left(\frac{1}{\alpha }\log\left(\frac{1+\alpha \left(1+e^{-\alpha }\right)}{1+\alpha \left(1+e^{-\alpha }\right)\mu_{F}\left(p\right)}\right)\right)}-1}{\alpha \left(1+e^{-\alpha }\right)}$$

(11) $$N\left(\mu_{F}\left(p\right)\right)=\frac{\left(1-\mu_{F}\left(p\right)\right)}{\left(1+\alpha \left(1+e^{-\alpha }\right)\mu_{F}\left(p\right)\right)},\alpha >0$$

Equation (11) is a fuzzy negation and it satisfies the following axioms:

(i) P1: Boundary conditions:

$$\mu_{F}\left(p\right)=1\begin{array}{c} \begin{array}{cc} \begin{array}{c} then \end{array} & N\left(1\right)=\frac{1-1}{1+\alpha \left(1+e^{-\alpha }\right)\times 1} \end{array} \end{array}=0$$

$$\mu_{F}\left(p\right)=0\begin{array}{c} \begin{array}{cc} then & N\left(0\right)=\frac{1-0}{1+\alpha \left(1+e^{-\alpha }\right)\times 0} \end{array} \end{array}=1$$

(ii) P2: Monotonicity

If $\mu_{F}(p)<\mu_{F}(q),$ then $N\left(\mu_{F}\left(p\right)\right)>N(\mu_{F}(q))$.

(iii) P3: Involution

$N\left(\mu_{F}\left(p\right)\right)$ is involutive that indicates $N\left(N\left(\mu_{F}\left(p\right)\right)\right)=\mu_{F}\left(p\right)$.

**Proof:** 
$$N\left(N\left(\mu_{F}\left(p\right)\right)\right)=\frac{1-N\left(\mu_{F}\left(p\right)\right)}{\left(1+\alpha \left(1+e^{-\alpha }\right)N\left(\mu_{F}\left(p\right)\right)\right)}=\frac{1-\frac{\left(1-\mu_{F}\left(p\right)\right)}{\left(1+\alpha \left(1+e^{-\alpha }\right)\mu_{F}\left(p\right)\right)}}{\left(1+\alpha \left(1+e^{-\alpha }\right)\frac{\left(1-\mu_{F}\left(p\right)\right)}{\left(1+\alpha \left(1+e^{-\alpha }\right)\mu_{F}\left(p\right)\right)}\right)}=\mu_{F}\left(p\right)$$

It can be noticed that if α=0, then $N\left(\mu_{F}\left(p\right)\right)=\left(1-\mu_{F}\left(p\right)\right)$; this is equivalent to standard Zadeh’s fuzzy complement. □

The intuitionistic fuzzy generator cannot be represented by all of the fuzzy complements. If the fuzzy complement satisfies the conditions, it will be referred to as an intuitionistic fuzzy generator:

$N\left(\mu_{F}\left(p\right)\right)=\left(1-\mu_{F}\left(p\right)\right)$ for all $\mu_{F}(p)\in \left[0,1\right]$, with N(0)=1 and N(1)=0.

The proposed fuzzy complement is the intuitionistic fuzzy generator and it satisfies the conditions. From the Equation (11), non-membership degree values are computed by using a new intuitionistic fuzzy generator, and new IFS (NIFS) becomes:

(12) $$F_{\alpha }^{NIFS}=\left\{p,\mu_{F}(p),v_{F}(p)=\frac{\left(1-\mu_{F}\left(p\right)\right)}{\left(1+\alpha \left(1+e^{-\alpha }\right)\mu_{F}\left(p\right)\right)}\left|p\in P\right.\right\}$$

and the hesitation degree can be represented as:

(13) $$\pi_{F}(p)=1-\mu_{F}(p)-v_{F}(p)$$

Equation (11), a new intuitionistic fuzzy generator, is used to expand and enhance the intensity levels over a range because some of the multimodal medical images are primarily dark. Varying the *α* value indicates a change in the intensity values not only in grayscale images but also a change in the ratio of components in the color images.

In image processing, entropy plays a significant role and is used to distinguish the texture of the image. The fuzzy entropy estimates ambiguity and fuzziness in a fuzzy set and was introduced by Zadeh. De Luca and S. Termini [41] introduced the first skeleton of non-parabolic entropy in 1972. Many researchers [42,43] have proposed various structures of entropy methods employing the IFS theory. In this article, a novel IFE function is presented, that can be determined as in [36], and it has been utilized to develop the proposed technique, which is described as:

(14) $$IFE\left(F;\alpha \right)=\frac{1}{n}\sum_{i=1}^{n}\pi_{F}\left(p_{i}\right)\exp\left(\pi_{F}(p_{i})\right)$$

where $\pi_{F}\left(p_{i}\right)=1-\mu_{F}\left(p_{i}\right)-v_{F}\left(p_{i}\right)$, $\mu_{F}\left(p_{i}\right)$, and $v_{F}\left(p_{i}\right)$ are the hesitation, membership, and non-membership degrees, respectively. Entropy (*IFE*) function is computed by using Equation (14) for the α values between [0.1–1.0], thus, it is optimized by calculating the highest entropy value using Equation (15), i.e.,

(15) $$\alpha_{opt}=\underset{\alpha }{max}\left(IFE\left(F;\alpha \right)\right)$$

With the known value of *α*, the membership values of the new intuitionistic fuzzy set (NIFS) are calculated, and finally, the new intuitionistic fuzzy image (NIFI) is represented below:

(16) $$F^{NIFI}=\left\{p,\mu_{F}(p;\alpha ),v_{F}(p;\alpha ),\pi_{F}\left(p;\alpha \right)\left|p\in P\right.\right\}$$

### 3.3. Intuitionistic Fuzzy Cross-Correlation (IFCC)

The cross-correlation of IFS [37] is a significant measure in IFS theory and has extraordinary fundamental potential in various areas, such as medical diagnosis, decision-making, recognition, etc. The IFCC function is used to measure the correlation between two intuitionistic fuzzy images (IFIs). Let $C_{1},C_{2}\in IFS\left(P\right)$ and $P=\left\{p_{1},p_{2},\ldots \ldots ,p_{n}\right\}$ be a finite universe of discourse, then the correlation coefficient is described as, follows:

(17) $$\rho^{*}\left(C_{1},C_{2}\right)=\frac{1}{2\times n}\sum_{g=1}^{n}\left[\alpha_{g}\left(1-\Delta \mu_{g}\right)+\beta_{g}\left(1-\Delta v_{g}\right)\right]\;\mathrm{where}\;\alpha_{g}=\frac{c-\Delta \mu_{g}-\Delta \mu_{max}}{c-\Delta \mu_{min}-\Delta \mu_{max}},\;\beta_{g}=\frac{c-\Delta v_{g}-\Delta v_{max}}{c-\Delta v_{min}-\Delta v_{max}},\;g=\left\{1,2,\ldots \ldots ,n\right\}\;\Delta \mu_{g}=\left|\mu_{C_{1}}\left(p_{g}\right)-\mu_{C_{2}}\left(p_{g}\right)\right|,\;\Delta v_{g}=\left|v_{C_{1}}\left(p_{g}\right)-v_{C_{2}}\left(p_{g}\right)\right|,\;\Delta \mu_{max}=\underset{g}{max}\left\{\left|\mu_{C_{1}}\left(p_{g}\right)-\mu_{C_{2}}\left(p_{g}\right)\right|\right\},\;\Delta \mu_{min}=\underset{g}{min}\left\{\left|\mu_{C_{1}}\left(p_{g}\right)-\mu_{C_{2}}\left(p_{g}\right)\right|\right\},\;\Delta v_{min}=\underset{g}{min}\left\{\left|v_{C_{1}}\left(p_{g}\right)-v_{C_{2}}\left(p_{g}\right)\right|\right\},\;\Delta v_{max}=\underset{g}{max}\left\{\left|v_{C_{1}}\left(p_{g}\right)-v_{C_{2}}\left(p_{g}\right)\right|\right\}$$

Here, the $\alpha_{g}\;and\;\beta_{g}$ and IFCC values range from [0–1], which depends on the constant value ‘*c*’.

## 4. Proposed Fusion Method

**Figure 1:**
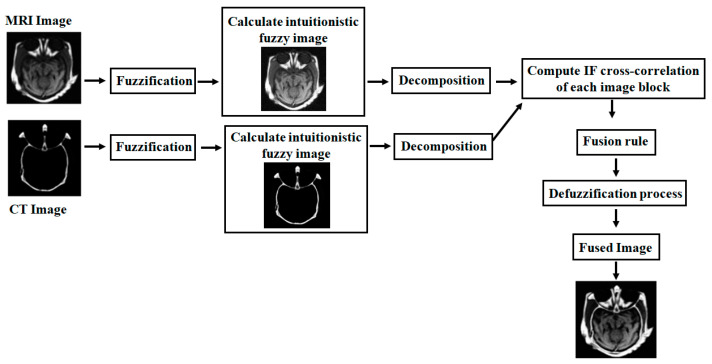
Flow chart of proposed grayscale medical image fusion algorithm.

**Figure 2:**
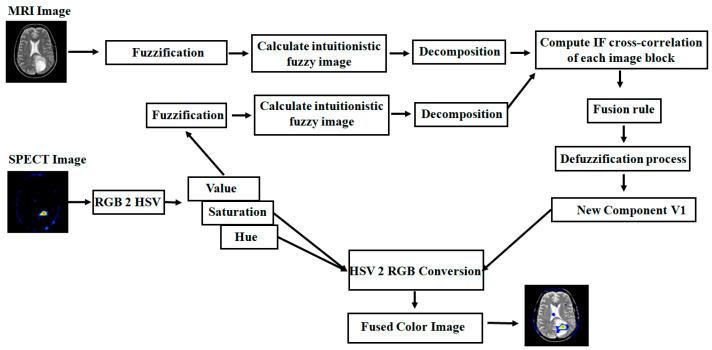
Flow chart of proposed color medical image fusion algorithm.

In this section, we present a new approach to IFS-based multimodality medical image fusion with the IFCC fusion rule. Here, various combinations of medical images are involved in the fusion process such as T1–T2 weighted MR images, T1-weighted MR–MRA images, MRI–CT images, MRI–PET images, and MR-T2–SPECT images. This proposed method can be implemented in both grayscale and color images. This fusion algorithm is arranged sequentially as shown in Figure 1 and Figure 2.

### 4.1. Grayscale Image Fusion Algorithm

1. Read the registered input images $I_{1}$ and $I_{2}$.
2. Initially, the first input image $I_{1}$ is fuzzified by using Equation (18):

(18) $$\mu_{I_{1}}\left(I_{gh1}\right)=\frac{I_{gh1}-I_{min}}{I_{max}-I_{min}}$$

where $I_{gh1}$ is the gray pixel of the first input image. $I_{max}\;\mathrm{and}\;I_{min}$ represent the highest and least gray level pixel values of the first input image, respectively.

3. Compute the optimum value, $\left(\alpha_{opt1}\right)$ for first input image by using IFE, which is given in Equations (14) and (15).
4. With the help of the optimized value, $\left(\alpha_{opt1}\right)$, calculate the fuzzified new IFI (NIFI) for the first input image by using Equations (19)–(22), which can be represented as $I_{IF1}$.

The membership degree of the NIFI is created as:

(19) $$\mu_{I_{1}}^{NIFS}\left(I_{gh1};\alpha_{opt1}\right)=\frac{\left(1+\alpha_{opt1}\left(1+e^{-\alpha_{opt1}}\right)\right)\mu_{I_{1}}\left(I_{gh1}\right)}{1+\alpha_{opt1}\left(1+e^{-\alpha_{opt1}}\right)\mu_{I_{1}}\left(I_{gh1}\right)}$$

Non-membership function is created as:

(20) $$\begin{array}{l} \nu_{I_{1}}^{NIFS}\left(I_{gh1};\alpha_{opt1}\right)=\frac{\left(1-\mu_{I_{1}}^{NIFS}\left(I_{gh1};\alpha_{opt1}\right)\right)}{\left(1+\alpha_{opt1}\left(1+e^{-\alpha_{opt1}}\right)\mu_{I_{1}}^{NIFS}\left(I_{gh1};\alpha_{opt1}\right)\right)}\\ =\frac{1-\mu_{I_{1}}\left(I_{gh1}\right)}{1+2\alpha_{opt1}\mu_{I_{1}}\left(I_{gh1}\right)\left(1+e^{-\alpha_{opt1}}\right)+\mu_{I_{1}}\left(I_{gh1}\right){\left[\alpha_{opt1}\left(1+e^{-\alpha_{opt1}}\right)\right]}^{2}} \end{array}$$

and finally, the hesitation degree is obtained as:

(21) $$\pi_{I_{1}}^{NIFS}\left(I_{gh1};\alpha_{opt1}\right)=1-\mu_{I_{1}}^{NIFS}\left(I_{gh1};\alpha_{opt1}\right)-v_{I_{1}}^{NIFS}\left(I_{gh1};\alpha_{opt1}\right)$$

(22) $$I_{IF1}=\left\{I_{gh1},\mu_{I_{1}}^{NIFS}\left(I_{gh1};\alpha_{opt1}\right),v_{I_{1}}^{NIFS}\left(I_{gh1};\alpha_{opt1}\right),\pi_{I_{1}}^{NIFS}\left(I_{gh1};\alpha_{opt1}\right)\right\}$$

5. Similarly, for the second input image, repeat from step 2 to step 4 to obtain the optimum value, $\alpha_{opt2}$, used to calculate NIFI ($I_{IF2}$):

(23) $$I_{IF2}=\left\{I_{gh2},\mu_{I_{2}}^{NIFS}\left(I_{gh2};\alpha_{opt2}\right),v_{I_{2}}^{NIFS}\left(I_{gh2};\alpha_{opt2}\right),\pi_{I_{2}}^{NIFS}\left(I_{gh2};\alpha_{opt2}\right)\right\}$$

6. Decompose the two NIFI images ($I_{IF1}$ and $I_{IF2}$) into small i×j blocks and the *k*^th^ block of two decomposed images are represented as $I_{IF1k}$ and $I_{IF2k}$, respectively.
7. Compute the intuitionistic fuzzy cross-correlation fusion rule between two windows of images ($I_{IF1k}$ and $I_{IF2k}$) and the *k*^th^ block of the fused $I_{IFk}$ image is obtained by using minimum, average, and maximum operations:

(24) $$I_{IFk}=\left\{\begin{array}{ccc} \min\left\{I_{IF1k},I_{IF2k}\right\} & if & \rho^{*}\left(I_{IF1k},I_{IF2k}\right)\leq 0\\ \frac{I_{IF1k}+I_{IF2k}}{2} & if & \rho^{*}\left(I_{IF1k},I_{IF2k}\right)=1\\ \max\left\{I_{IF1k},I_{IF2k}\right\} & . & otherwise \end{array}\right.$$

8. Reconstruct the fused IFI image by the combined small blocks.
9. Finally, the fused image can be obtained in the crisp domain by using the defuzzification process, which is obtained by the inverse function of Equation (18).

(25) $$F_{c}\left(i,j\right)=\left(\left(I_{max}-I_{min}\right)*I_{IFk}+I_{min}\right)$$

### 4.2. Color Image Fusion Algorithm

The complete fusion algorithm for the combination of gray (MRI) and color images (PET/SPECT) is arranged sequentially as shown in Figure 2.

1. Consider MRI and PET/SPECT as input images. The PET/SPECT image is converted into an HSV color model, such as hue (*H*), saturation (*S*), and value (*V*).
2. For the fusion process, take the MRI image and *V* component image, and then perform a grayscale image fusion algorithm from step 2 to step 9 as shown in Section 4.1, to get the fused component (*V*_1_).
3. Finally, the colored fused image can be obtained by considering the brightness image (*V*_1_) and unchanged hue (*H*) and saturation (*S*) parts and then converting into the RGB color model.

## 5. Experimental Results and Discussion

This section represents a brief explanation of the effectiveness of the proposed method and a detailed comparison of various existing algorithms with the help of performance metrics. In this paper, all input medical images are assumed to be perfectly registered, and experiments are performed with two different modalities of medical images, where the data is collected and downloaded from metapix and whole brain atlas [44,45]. The fusion of these two modalities of the medical image will provide a composite image, which will be more useful for diagnosing diseases, tumors, lesion locations, etc. 

In this article, we have performed a new intuitionistic fuzzy set-based image fusion over various modalities of medical image datasets of dimensions 256×256 using the IFCC fusion rule. The proposed fusion algorithm is used to expand and enhance the intensity levels over a range because some of the medical images are primarily dark. Varying the *α* value indicates a change not only in the intensity values but also changes in the ratio of components in the color image. These enhanced medical images are fused to obtain a single image with more complementary information and better quality. Hence, we conclude that a single medical image cannot provide the required information regarding the disease. As a result, MIF is required to obtain all relevant and complete information in a single resultant image.

The evaluation of the fused image can be completed with the help of subjective (visual) and objective (quantitative) analysis, respectively. The subjective analysis is performed with the visual appearance, and the objective analysis is finished with a set of performance metrics. In this paper, eight metrics are used: API [46], SD [46], AG [47], SF [48], MSF [49], CC [50], MI [51], and FS [48].

The input images are $I_{1}\left(g,h\right)$ and $I_{2}\left(g,h\right)$ and the fused image is $Fused\left(g,h\right)$ with G×H dimensionality.

➢ *API*: *API* is used to quantify the average intensity values of the fused image i.e., brightness, which can be defined as:

(26) $$API=\frac{1}{G\times H}\sum_{g=1}^{G}\sum_{h=1}^{H}\left[Fused\left(g,h\right)\right]$$

➢ *SD*: *SD* is used to represent the amounts of intensity variations—contrast—in an image. It is described as

(27) $$SD=\sqrt{\frac{1}{G\times H}\sum_{g=1}^{G}\sum_{h=1}^{H}{\left[Fused\left(g,h\right)-\mu \right]}^{2}}$$

➢ *AG*: This metric is used to measure the sharpness degree and clarity, which is represented as:

(28) $$AG=\frac{1}{\left(G-1\right)\left(H-1\right)}\sum_{g=1}^{G-1}\sum_{h=1}^{H-1}\frac{\sqrt{{\left[Fused(g,h)-Fused(g+1,h)\right]}^{2}+{\left[Fused(g,h)-Fused(g,h+1)\right]}^{2}}}{\sqrt{2}}$$

➢ *SF*: *SF* reflects the rate of change in the gray level of the image and also measures the quality of the image. For better performance, the SF value should be high. It can be calculated as follows:

(29) $$SF=\sqrt{{\left(RF\right)}^{2}+{\left(CF\right)}^{2}}$$

where

$$RF=\sqrt{\frac{1}{G\times H}\sum_{g=1}^{G}\sum_{h=2}^{H}{\left[Fused\left(g,h\right)-Fused\left(g,h-1\right)\right]}^{2}}\;CF=\sqrt{\frac{1}{G\times H}\sum_{g=2}^{G}\sum_{h=1}^{H}{\left[Fused\left(g,h\right)-Fused\left(g-1,h\right)\right]}^{2}}$$

➢ *MSF*: This metric is used to measure the overall active levels present in the fused image. It can be employed as follows:

(30) $$MSF=\sqrt{{\left(RF_{F}\right)}^{2}+{\left(CF_{F}\right)}^{2}+{\left(DF_{F}\right)}^{2}},\;DF_{F}=A+B$$

where

$$RF_{F}=\sqrt{\frac{1}{G\times \left(H-1\right)}\sum_{g=1}^{G}\sum_{h=2}^{H}{\left[Fused\left(g,h\right)-Fused\left(g,h-1\right)\right]}^{2}}\;CF_{F}=\sqrt{\frac{1}{\left(G-1\right)\times H}\sum_{g=2}^{G}\sum_{h=1}^{H}{\left[Fused\left(g,h\right)-Fused\left(g-1,h\right)\right]}^{2}}\;A=\sqrt{\frac{1}{\left(G-1\right)\times \left(H-1\right)}\sum_{g=2}^{G}\sum_{h=2}^{H}{\left[Fused\left(g,h\right)-Fused\left(g-1,h-1\right)\right]}^{2}}\;B=\sqrt{\frac{1}{\left(G-1\right)\times \left(H-1\right)}\sum_{g=2}^{G}\sum_{h=2}^{H}{\left[Fused\left(g-1,h\right)-Fused\left(g,h-1\right)\right]}^{2}}$$

➢ *CC*: This metric represents the similarity between the source and fused images. The range of *CC* is [0–1]. For high similarity, the *CC* value is 1 and it decreases as the dissimilarity increases. It is represented as follows:

(31) $$CC=\frac{CC_{I_{1},Fused}+CC_{I_{2},Fused}}{2}$$

where

$$CC_{I_{1},Fused}=\frac{\sum_{g=1}^{G}\sum_{h=1}^{H}\left(I_{1gh}-\mu_{I_{1gh}}\right)\left(Fused_{gh}-\mu_{Fused}\right)}{\sqrt{\left(\sum_{g=1}^{G}\sum_{h=1}^{H}{\left(I_{1gh}-\mu_{I_{1gh}}\right)}^{2}\right)\left(\sum_{g=1}^{G}\sum_{h=1}^{H}{\left(Fused_{gh}-\mu_{Fused}\right)}^{2}\right)}},\;CC_{I_{2},Fused}=\frac{\sum_{g=1}^{G}\sum_{h=1}^{H}\left(I_{2gh}-\mu_{I_{2gh}}\right)\left(Fused_{gh}-\mu_{Fused}\right)}{\sqrt{\left(\sum_{g=1}^{G}\sum_{h=1}^{H}{\left(I_{2gh}-\mu_{I_{2gh}}\right)}^{2}\right)\left(\sum_{g=1}^{G}\sum_{h=1}^{H}{\left(Fused_{gh}-\mu_{Fused}\right)}^{2}\right)}}$$

➢ *MI*: The *MI* parameter is used to calculate the total information that is transferred to the fused image from input images.

(32) $$MI_{T}=MI_{I_{1},Fused}+MI_{I_{2},Fused}$$

where $MI_{I_{1},Fused}=\sum_{g=1}^{g}\sum_{h=1}^{h}h_{I_{1},Fused}(g,h)\log_{2}\left[\frac{h_{I_{1},Fused}(g,h)}{h_{I_{1}}(g,h)h_{Fused}(g,h)}\right]$ is the MI of input $I_{1}\left(g,h\right)$ and fused images, and 

$MI_{I_{2},Fused}=\sum_{g=1}^{g}\sum_{h=1}^{h}h_{I_{2},Fused}(g,h)\log_{2}\left[\frac{h_{I_{2},Fused}(g,h)}{h_{I_{2}}(g,h)h_{Fused}(g,h)}\right]$ is the MI of input $I_{2}\left(g,h\right)$ and fused images, respectively. For better performance, the MI value should be high.

➢ *FS*: *FS* is introduced to measure the symmetry of the fused image with respect to the source images. If the value of *FS* is close to 2, this indicates both input images equally contribute to the fused image. Therefore, the fused image quality will be better.

(33) $$FS=2-\left|\left(\frac{MI_{I_{1},Fused}}{MI}-0.5\right)\right|$$

### 5.1. Subjective-Type Evaluation

**Figure 3:**
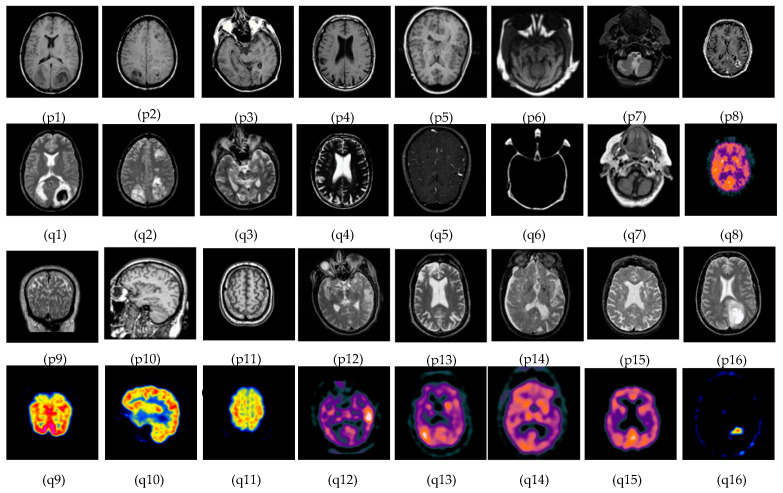
Medical image datasets: (**p1**–**p4**) and (**q1**–**q4**) are MR T1–MR T2 input images: (**p5**) and (**q5**) are T1 weighted MR–MRA input images; (**p6**,**p7**) and (**q6**,**q7**) are MRI–CT input images; (**p8**–**p11**) and (**q8**–**q11**) are MRI–PET input images; and (**p12**–**p16**) and (**q12**–**q16**) are the MR-T2–SPECT input images.

**Figure 4:**
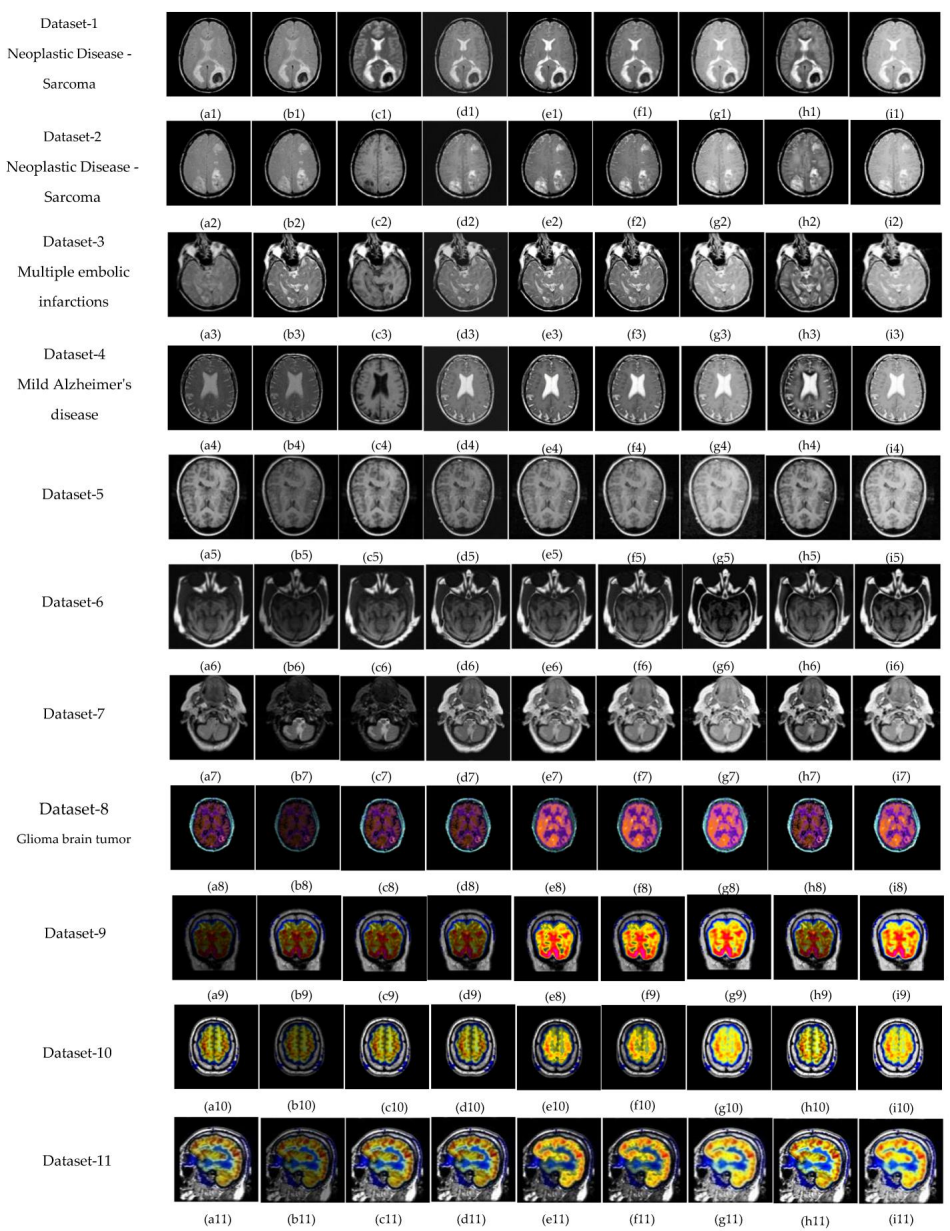

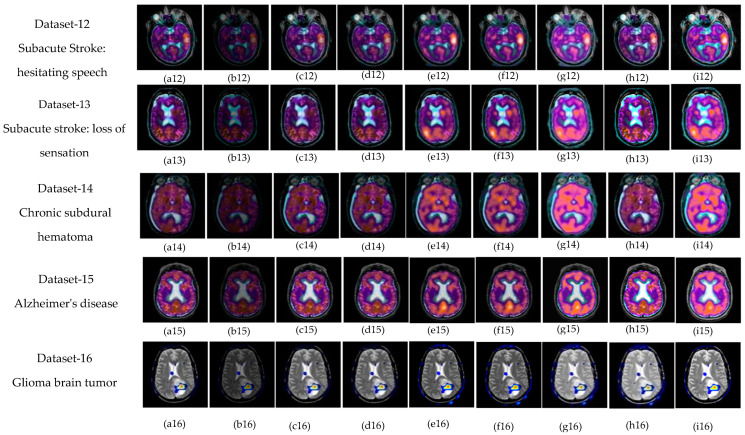
Fused images using: (**a**) PCA method, (**b**) DWTPCA [52] method, (**c**) DWT + fuzzy method [29], (**d**) Contourlet transform (CONT) based method, (**e**) Chaira’s IFS [53], (**f**) Bala’s IFS [54], (**g**) Sugeno’s IFS [55], (**h**) PC-NSCT method [56], and (**i**) Proposed method.

The subjective evolution is carried out on various input datasets as shown in Figure 3. In this paper, five groups of datasets have been used. The group 1 input images are MR-T1–MR-T2 datasets as shown in Figure 3((p1–p4) and (q1–q4)). Group 2 input images are MR-T1 and MRA as shown in Figure 3((p5) and (q5)). Group 3 input images are MRI and CT in Figure 3((p6–p7) and (q6–q7)), and group 4 input data set images are MRI and PET in Figure 3((p8–p11) and (q8–q11)). Finally, group 5 input images are MR-T2 and SPECT datasets as shown in Figure 3((p12–p16) and (q12–q16)). In this article, the performance of the proposed fusion scheme is compared with various existing algorithms, namely, the PCA method, Naidu’s [52] method, Sanjay’s [29] method, contourlet transform (CONT) method, Chaira’s IFS [53] method, Bala’s IFS [54] method, Sugeno’s IFS [55] method, and Zhu’s [56] method are in Figure 4. The fusion results of the PCA method-based fusion images are shown in the first column in Figure 4(a1–a16), DWTPCA method-based fusion images are displayed in the second column in Figure 4(b1–b16), DWT with fuzzy method-based fusion images are shown in the third column in Figure 4(c1–c16), CONT method based fusion images are displayed in the fourth column in Figure 4(d1–d16), Chaira’s IFS-method based fusion images are shown in the fifth column in Figure 4(e1–e16), Bala’s IFS method based fusion images are displayed in the sixth column in Figure 4(f1–f16), Sugeno’s IFS-method based fusion images in the seventh column in Figure 4(g1–g16), PC- NSCT method based fusion images are in the eighth column in Figure 4(h1–h16). Finally, the proposed fusion images are exhibited in the last column in Figure 4(i1–i16). Subjective analysis is related to human perception, and the proposed fusion method proves, the fused image has greater contrast, luminance, and better edge information than other existing methods, and clear tumor regions are shown in Figure 4((i4), (i8), (i12), (i13), and (i16)).

The proposed fusion results show that the quality of the fused image is better than other existing fusion methods. Among all the groups of medical image datasets, the first group of medical image datasets are T1–T2 weighted MR images. Fusing these two images shows soft tissue and an enhanced tumor region. The second group of medical image datasets are MR-T1and MRA images. MR-T1 images produce delicate tissue data but do not detect the abnormalities in the image, while the MRA image easily detects the abnormalities but due to low spatial resolution, is unable to produce the tissue information. Fusion of these images (MR-T1 and MRA) shows the complementary information with detailed lesion locations in the fused image. 

The third group dataset consists of MRI and CT images, which are taken from reference [44]. MRI imaging produces delicate tissue data, while CT imaging gives bone information. The combination of these two images produces a quality fused image, which will be more useful for the diagnosis of disease. The fourth and fifth medical image datasets are MRI–PET and MR-T2–SPECT images. The fusion of these combinations to get more complementary information is achieved in a fused image and highlights the tumor regions, which will be helpful for medical-related problems.

### 5.2. Objective Evaluation

**Figure 5:**
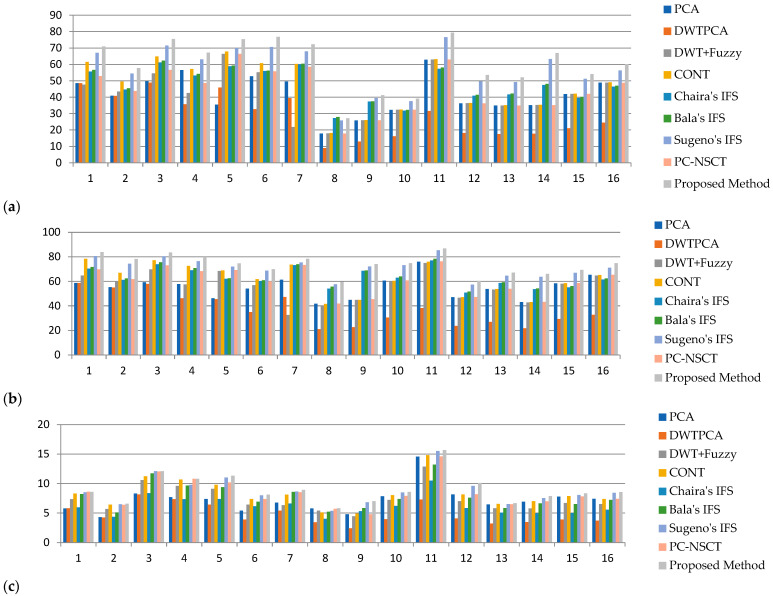
Graphical representation of (**a**) API, (**b**) SD, (**c**) AG measures of proposed and other existing methods.

The fused image quality cannot be completely judged by subjective analysis. Therefore, objective evaluation is preferable for better analysis of fused images using various quality metrics. The proposed method and other existing methods’ results are listed in Table 2, Table 3, Table 4, Table 5, Table 6, Table 7, Table 8 and Table 9. The values of the average pixel intensity (API) are tabulated in Table 2. It can be observed that the proposed fusion method provides the highest API values, which indicates that the fused image has good quality. The graphical representations of API values are shown in Figure 5a. The standard deviation quantity values are tabulated in Table 3. It can be shown that the proposed method’s SD values are greater than the other existing techniques, which indicates the output fused image has better texture details and is graphically presented in Figure 5b.

The average gradient (AG) values are shown in Table 4. It can be seen that the proposed method gives the highest AG values, which reveals that more complementary information is presented in a fused image, and this is presented graphically in Figure 5c.

**Figure 6:**
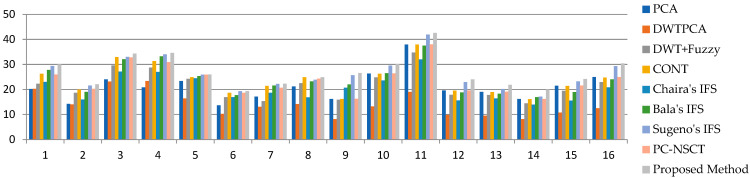
Graphical representation of SF measures of proposed and existing methods.

**Figure 7:**
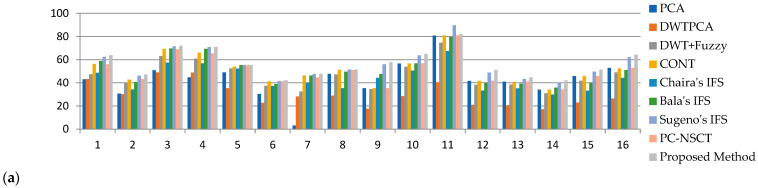

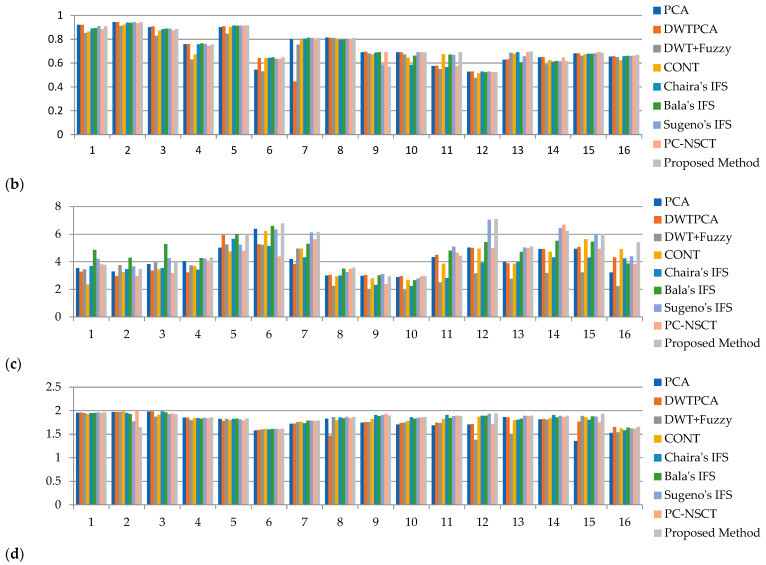
Graphical representation of (**a**) MSF, (**b**) CC, (**c**) MI, and (**d**) FS measures of proposed and existing methods.

The SF values are listed in Table 5. It can be seen that the SF of the proposed method gives superior values to the other methods, which indicates texture changes and detailed differences are reflected in a fused image, and this is shown graphically in Figure 6. The MSF values are listed in Table 6. It can be seen that the MSF values of the proposed method provides greater values than the other methods, which indicates that a fused image has more detailed information, and this is observed graphically in Figure 7a.

The CC, MI, and FS values of all datasets and existing fusion methods are listed in Table 7, Table 8 and Table 9. In the proposed fusion method, the average values of CC, MI, and FS values are better, and some datasets are moderate, which shows that the proposed fused image has more information and symmetry. The graphical representation of CC, MI, and FS is shown in Figure 7b–d.

### 5.3. Ranking Analysis

In this article, the proposed intuitionistic fuzzy set based multimodal medical image fusion algorithm provides better results than other methods using various quality metrics. Objective evaluation was used in Section 5.2. This showed the ranking analysis of each method based on the average value of each quality metric, as shown in Table 10. The best performance of the fusion method was ranked 1, and the worst performance of the fusion method was ranked 9.

### 5.4. Running Time

The computational efficiency of the proposed and existing medical image fusion methods such as PCA, DWT, Contourlet, DWT + fuzzy, Chaira’s IFS, Bala’s IFS, Sugeno’s IFS, and PC-NSCT are shown in Table 11. Compared with all methods, the DWTPCA method takes the least execution time of 0.60 s because the image pixels are directly selected. Hence, it is found that the DWTPCA fusion method performance is poor in terms of subjectivity and objectivity. The highest execution time of the fusion method was PC-NSCT, which was 36.72 s due to decomposition levels and fusion rules. The second highest execution time of the Contourlet transforms method was 17.29 s. The third-highest execution time of the DWT + fuzzy method was 1.48 s. The average running time of the proposed method was 1.19. However, the proposed method provides better performance with relatively low execution times and less complexity than the other methods.

## 6. Conclusions

In this article, a novel IFS-based medical image fusion process was proposed, which included four steps. Firstly, the registered input images were fuzzified. Secondly, intuitionistic fuzzy images were created by the optimum value, α using IFE. Thirdly, a fused IFI image was obtained using the IFCC fusion rule with block processing. Fourthly, the defuzzification operation was performed for the final enhanced fused image. This method is an extension of the various existing methods, such as PCA, DWTPCA, DWT + Fuzzy, CONT, Chaira’s IFS, Bala’s IFS, Sugeno’s IFS, and PC-NSCT. These existing algorithms do not provide a quality fused image, and include various drawbacks, such as blocking artifacts, poor visibility of tumor regions, invisible blood vessels, low contrast, and vague boundaries. This proposed method overcomes the difficulties present in the existing methods and provides a better enhanced fused image without uncertainties. 

The experimental result shows that the proposed fusion method gives a better fusion performance in terms of subjective and objective analysis, respectively. In Figure 4(i4), the soft tissue and tumor regions are clearly enhanced and the obtained SD (79.83) and SF (34.60) values are large in Table 3 and Table 5, respectively. In Figure 4(i5), the soft tissue and lesion structure information are reflected exactly in a fused image, and the obtained quantitative value is 75.38, as shown in Table 2. In Figure 4(i8), the anatomy and functional information are visible with high quality in a fused image, and the quantitative values attained show that SD, AG, SF, MSF, MI, and FS are higher (59.54, 5.80, 24.92, 51.53, 3.5689, 1.8658) in Table 3, Table 4, Table 5, Table 6, Table 7, Table 8 and Table 9. In Figure 4(i16), the tumor region was clearly enhanced, and attained high performance metric values compared to the other existing fusion methods. As previously discussed, the heart of this proposed fusion algorithm is to calculate the intuitionistic fuzzy membership function, which is obtained by the optimum value, α using IFE. For better diagnosis and superior outcomes, the proposed fusion method can be extended to fuse different medical datasets based on the advanced fuzzy sets, such as the neutrosophic fuzzy set, pythagorean fuzzy set and fusion rules.

## Figures and Tables

**Table 1:** Comparison of the existing fusion methods.

Fusion Methods	Modalities	Merits	Demerits
IHS and PCA	MRI-PET	Good spatial features and better color visualization in a fused image.	Low contrast and distorted boundaries.
Pyramid	MRI-CT	Preserves better outlines in the fused image.	Due to a lack of spatial orientation selectivity, the unwanted edges and blocking effects exist in the fused image.
SVD	MRI-CT	Provides better quality fused image.	Fails to show the clear boundaries of the tumor region.
DWT	MRI-CT, MRI-PET	Provides good localization in both time and frequency.	Has more complexity and lack of edges information.
CONT	MRI-CT	Fused image has better edges and is superior to DWT and Curvelet transform.	Does not provide the shift invariance, may cause blocking effects
NSCT	MRI-CT	Superior to traditional transform techniques in terms of directionality.	Complexity is high.
NSST	MRI-CT	Fusion process is superior to NSCT with lower complexity.	Low brightness and contrast due to uncertainties, and high computational time.

**Table 2:** Performance evaluation of the fusion methods using the API measure.

Medical Image Modality		Fusion Techniques								
	Data Sets	PCA	DWTPCA	DWT + Fuzzy	CONT	Chaira’s IFS	Bala’s IFS	Sugeno’s IFS	PC-NSCT	Proposed Method
MR T1–MR T2	1	48.53	48.55	47.66	61.52	55.65	56.62	67.11	52.92	70.9
	2	40.94	40.79	43.4	49.59	44.69	45.4	54.38	43.77	57.77
	3	49.77	48.83	54.58	64.85	61.23	62.3	71.51	56.63	75.53
	4	56.54	35.73	42.57	57.2	53.15	54.23	63.11	48.66	67.15
MR-T1–MRA	5	35.5	45.87	66.38	67.85	58.82	59.34	69.92	66.38	75.38
MRI–CT	6	52.74	32.67	55.24	60.79	55.99	56.23	70.58	55.77	76.85
	7	49.54	39.47	21.87	60.33	59.92	60.36	67.93	58.52	72.23
MRI–PET	8	17.86	9.01	17.92	18.16	27.29	27.96	25.85	17.89	27.17
	9	25.81	13.01	25.92	26.04	37.31	37.45	39.41	25.88	41.21
	10	32.24	16.21	32.33	32.49	31.85	32.21	37.54	32.33	39.09
	11	62.82	31.56	62.98	63.22	57.32	58.07	76.55	62.96	79.4
MR-T2–SPECT	12	36.24	18.22	36.34	36.42	40.9	41.47	49.69	36.28	53.57
	13	34.87	17.54	34.96	35.11	41.7	42.18	49.21	34.95	52.07
	14	35.12	17.71	35.25	35.37	47.41	48.07	63.28	35.14	66.98
	15	41.89	21.06	42	42.15	39.6	40.24	51.23	42.01	54.05
	16	48.85	24.44	48.87	49.11	46.44	46.95	56.3	48.78	60.14
**Average Value**		41.83	28.79	41.77	47.51	47.45	48.07	57.10	44.93	**60.59**

**Table 3:** Performance evaluation of the fusion methods using SD measures.

Medical Image Modality		Fusion Techniques								
	Data Sets	PCA	DWTPCA	DWT + Fuzzy	CONT	Chaira’s IFS	Bala’s IFS	Sugeno’s IFS	PC-NSCT	Proposed Method
MR T1–MR T2	1	58.73	58.71	64.74	78.34	70.36	71.75	80.51	69.79	83.83
	2	55.21	55.02	60.17	67.04	61.2	62.32	74.36	61.79	78.16
	3	59.25	57.86	69.74	77.06	73.98	75.53	80.43	73.08	83.45
	4	57.79	46.16	57.5	72.55	69.02	70.84	76.46	68.38	79.83
MR-T1–MRA	5	46.19	45.49	68.52	68.86	62.11	62.45	72.01	69.22	74.73
MRI–CT	6	54.1	34.95	56.9	61.73	60.37	60.89	68.77	60.03	69.87
	7	61.41	47.21	32.58	73.7	73.22	73.88	75.45	73.42	78.27
MRI–PET	8	41.83	21.04	40.47	41.61	54.01	55.71	57.6	41.98	59.54
	9	44.92	22.61	44.84	44.89	68.6	68.87	72.23	45.46	74.12
	10	60.57	30.43	59.91	60.4	63.04	64.05	73.13	60.74	74.9
	11	75.98	38.16	75.01	75.93	76.97	78.37	85.24	76.18	86.72
MR-T2–SPECT	12	47.11	23.67	46.61	47.13	50.84	51.69	57.39	47.24	60.38
	13	53.72	26.98	53.33	53.75	58.7	59.33	64.67	53.85	67.1
	14	43.1	21.7	42.8	43.16	53.66	54.18	63.68	43.30	66.03
	15	58.44	29.36	58.03	58.49	55.09	56.19	66.97	58.60	69.4
	16	65.31	32.68	64.74	65.15	61.47	62.38	71.09	65.39	74.78
**Average Value**	-	55.23	37.00	55.99	61.85	63.29	64.28	71.25	60.53	**73.82**

**Table 4:** Performance evaluation of the fusion methods using the AG measure.

Medical Image Modality		Fusion Techniques								
	Data Sets	PCA	DWTPCA	DWT + Fuzzy	CONT	Chaira’s IFS	Bala’s IFS	Sugeno’s IFS	PC-NSCT	Proposed Method
MR T1–MR T2	1	5.79	5.8	7.35	8.31	5.96	8.21	8.51	8.62	8.6
	2	4.29	4.25	5.7	6.42	4.37	5.09	6.48	6.4	6.59
	3	8.32	8.15	10.6	11.25	8.38	11.73	12.1	12.05	12.1
	4	7.68	7.36	9.59	10.69	7.35	9.65	9.8	10.81	10.81
MR-T1–MRA	5	7.4	6.43	9.11	9.78	7.37	9.38	11.03	10.18	11.33
MRI–CT	6	5.4	3.9	6.43	7.39	6.15	6.92	7.99	7.39	8.12
	7	6.77	5.4	6.33	8.12	6.63	8.6	8.64	8.54	8.94
MRI–PET	8	5.78	3.45	5.42	5.10	4.04	5.24	5.37	5.73	5.80
	9	4.79	2.41	4.47	4.91	5.31	5.83	6.84	4.8	7.01
	10	7.86	3.96	7.24	8.04	6.22	7.37	8.5	7.92	8.58
	11	14.55	7.3	12.88	14.81	10.49	13.2	15.53	14.59	15.66
MR-T2–SPECT	12	8.15	4.09	7.03	8.17	5.85	7.59	9.59	8.18	10.09
	13	6.47	3.24	5.8	6.54	5.06	5.85	6.52	6.49	6.69
	14	6.93	3.48	5.79	7	4.97	6.65	7.55	6.97	7.88
	15	7.79	3.91	6.7	7.87	5.02	6.51	8.06	7.82	8.34
	16	7.41	3.71	6.52	7.37	5.58	7.23	8.43	7.42	8.57
**Average Value**	-	6.75	4.57	6.90	7.78	5.82	7.36	8.28	7.91	**8.53**

**Table 5:** Performance evaluation of the fusion methods using the SF measure.

Medical Image Modality		Fusion Techniques								
	Data Sets	PCA	DWTPCA	DWT + Fuzzy	CONT	Chaira’s IFS	Bala’s IFS	Sugeno’s IFS	PC-NSCT	Proposed Method
MR T1–MR T2	1	20.18	20.28	22.27	26.25	22.99	27.81	29.42	26.01	30.04
	2	14.23	14.02	18.64	19.99	15.95	19	21.55	20.23	22.05
	3	24.04	23.18	29.62	32.96	27.17	32.05	33	32.79	34.3
	4	20.82	23.36	28.76	31.37	26.98	33.25	34.05	30.9	34.6
MR-T1–MRA	5	23.41	16.43	24.23	24.88	24.48	25.36	25.94	25.92	25.98
MRI–CT	6	13.69	10.25	16.91	18.65	16.9	17.69	19.3	18.67	19.3
	7	17.16	13.07	15.31	21.45	18.68	21.55	22.19	20.66	22.27
MRI–PET	8	21.22	14.16	22.49	24.9	16.85	23.23	23.91	24.31	24.92
	9	16.2	8.14	15.88	16.17	20.71	22.03	25.71	16.26	26.54
	10	26.32	13.2	24.85	26.28	23.63	26.48	29.53	26.4	30.13
	11	37.92	19.02	34.77	37.93	31.99	37.49	41.97	38.01	42.6
MR-T2–SPECT	12	19.57	9.82	17.85	19.56	15.64	18.84	22.93	19.62	24.02
	13	19.04	9.55	17.76	18.97	16.41	18.28	20.2	19.08	21.84
	14	16.17	8.12	14.47	16.15	13.97	16.94	17.12	16.23	19.96
	15	21.52	10.79	19.43	21.44	15.54	18.97	23.24	21.57	24.16
	16	24.96	12.49	22.91	24.76	20.87	24.02	29.35	24.96	30.41
**Average Value**	-	21.03	14.12	21.63	23.86	20.55	23.94	26.21	23.85	**27.07**

**Table 6:** Performance evaluation of the fusion methods using the MSF measure.

Medical Image Modality		Fusion Techniques								
	Data Sets	PCA	DWTPCA	DWT + Fuzzy	CONT	Chaira’s IFS	Bala’s IFS	Sugeno’s IFS	PC-NSCT	Proposed Method
MR T1–MR T2	1	43.01	43.22	47.43	56.27	48.63	58.86	62.39	55.86	63.79
	2	30.58	30.14	40.21	42.72	34.21	40.66	46.16	43.29	47.23
	3	50.81	49.05	63.05	69.36	57.43	69.59	71.51	69.04	72.04
	4	44.77	48.87	60.89	66.10	56.75	69.33	70.96	65.35	71.08
MR-T1–MRA	5	48.95	35.35	52.49	53.99	52.28	55.25	55.36	55.12	55.45
MRI–CT	6	30.36	22.62	37.41	41.13	37.16	38.95	41.47	41.24	42.30
	7	3.07	28.13	32.61	46.26	40.21	46.34	47.59	44.64	47.75
MRI–PET	8	47.80	29.00	47.31	51.09	35.36	49.67	51.49	50.98	51.53
	9	35.28	17.73	34.62	35.28	44.21	47.49	56.00	35.42	57.81
	10	56.70	28.44	53.89	56.69	50.47	56.82	63.72	56.86	64.95
	11	80.76	40.51	74.61	80.86	67.51	79.53	89.72	80.97	82.04
MR-T2–SPECT	12	41.64	20.88	38.28	41.61	33.35	40.10	48.75	41.75	50.99
	13	41.02	20.57	38.50	40.85	35.26	39.29	43.38	41.12	44.71
	14	34.14	17.14	30.96	34.10	29.82	35.80	40.35	34.26	42.02
	15	45.80	22.97	41.75	45.61	33.14	40.25	49.54	45.92	51.48
	16	52.94	26.48	48.95	52.46	44.21	50.98	62.13	52.94	64.32
**Average Value**	-	42.98	30.07	46.44	50.90	43.75	51.18	56.28	50.92	**56.84**

**Table 7:** Performance evaluation of the fusion methods using the CC measure.

Medical Image Modality		Fusion Techniques								
	Data Sets	PCA	DWTPCA	DWT + Fuzzy	CONT	Chaira’s IFS	Bala’s IFS	Sugeno’s IFS	PC-NSCT	Proposed Method
MR T1–MR T2	1	0.92	0.9201	0.8523	0.8647	0.8905	0.8932	0.9093	0.8838	0.9089
	2	0.9428	0.9433	0.9117	0.9214	0.9392	0.9381	0.9433	0.9323	0.9421
	3	0.9007	0.9064	0.8286	0.8725	0.8844	0.889	0.8892	0.8715	0.8849
	4	0.7583	0.7587	0.6296	0.6721	0.7572	0.7659	0.7585	0.7444	0.7553
MR-T1–MRA	5	0.9012	0.9078	0.8457	0.9021	0.9152	0.9134	0.9129	0.9133	0.9157
MRI–CT	6	0.5444	0.6413	0.5305	0.6412	0.6439	0.6472	0.635	0.6348	0.6481
	7	0.8007	0.445	0.7548	0.7951	0.8055	0.8127	0.8102	0.8043	0.8111
MRI–PET	8	0.8132	0.8106	0.8116	0.7985	0.794	0.7951	0.7951	0.8034	0.8088
	9	0.6912	0.6954	0.6795	0.6715	0.688	0.6901	0.5878	0.6936	0.5694
	10	0.691	0.6912	0.6724	0.6457	0.585	0.6609	0.6907	0.6911	0.6886
	11	0.574	0.5755	0.5494	0.6736	0.5662	0.6709	0.6695	0.5736	0.6907
MR-T2–SPECT	12	0.5279	0.5296	0.4755	0.5137	0.5285	0.5228	0.5288	0.5236	0.5226
	13	0.6283	0.6313	0.6858	0.6783	0.6906	0.6067	0.6567	0.6925	0.6966
	14	0.6476	0.6513	0.5901	0.6238	0.6112	0.6167	0.6137	0.6456	0.6165
	15	0.681	0.6819	0.6618	0.6736	0.6778	0.677	0.6819	0.6932	0.6867
	16	0.6541	0.6574	0.6495	0.6219	0.6567	0.6595	0.6595	0.662	0.6693
**Average Value**	-	0.7298	0.7154	0.6956	0.7231	0.7271	0.735	0.7339	0.7352	**0.7385**

**Table 8:** Performance evaluation of the fusion methods using the MI measure.

Medical Image Modality		Fusion Techniques								
	Data Sets	PCA	DWTPCA	DWT + Fuzzy	CONT	Chaira’s IFS	Bala’s IFS	Sugeno’s IFS	PC-NSCT	Proposed Method
MR-T1–MR-T2	**1**	3.5405	3.2795	3.4464	2.3495	3.6865	4.8538	4.2121	3.8146	3.7825
	2	3.2935	2.9621	3.7514	3.2415	3.4637	4.292	3.6564	2.9574	3.4782
	3	3.837	3.3622	3.9574	3.4521	3.5411	5.2866	4.2527	3.1686	3.9925
	4	4.0495	3.2325	3.7457	3.6848	3.4302	4.2621	4.2265	4.0854	4.3005
MR-T1–MRA	5	5.0121	5.9402	5.2496	4.7354	5.6626	5.9854	5.2456	4.791	5.9928
MRI–CT	6	6.3918	5.2744	5.2314	6.2198	5.1325	6.5985	6.3305	4.3827	6.7901
	7	4.2013	3.851	4.9572	4.947	4.3207	5.2971	6.1245	5.6228	6.165
MRI–PET	8	3.0026	3.0452	2.2536	2.9358	3.0057	3.5067	3.2504	3.4873	3.5689
	9	2.9769	3.0468	1.9956	2.785	2.3185	3.0095	3.1047	2.3645	2.9308
	10	2.8845	2.9636	1.9311	2.6831	2.2413	2.6624	2.7858	2.9416	2.9711
	11	4.3382	4.51	2.4966	3.8536	2.8213	4.8101	5.0956	4.6593	4.4321
MR-T2–SPECT	12	5.0262	5.0045	3.1563	4.9574	3.9424	5.4231	7.0542	4.9962	7.1046
	13	3.9957	3.8844	2.76	3.8614	3.9952	4.7176	5.027	4.9831	5.1158
	14	4.9323	4.9244	3.1878	4.7164	4.3146	5.5147	6.4363	6.6907	6.2446
	15	4.934	5.0671	3.2207	5.6416	4.3017	5.4551	6.0178	4.9347	6.0238
	16	3.2219	4.3176	2.2222	4.9135	4.2378	3.8704	4.394	3.8471	5.416
**Average Value**	-	4.1024	4.0416	3.3477	4.0611	3.776	4.7216	4.8259	4.2329	**4.8943**

**Table 9:** Performance evaluation of the fusion methods using the FS measure.

Medical Image Modality		Fusion Techniques								
	Data Sets	PCA	DWTPCA	DWT + Fuzzy	CONT	Chaira’s IFS	Bala’s IFS	Sugeno’s IFS	PC-NSCT	Proposed Method
MR T1–MR T2	1	1.9552	1.9624	1.9516	1.9254	1.9515	1.9537	1.9597	1.9524	1.9655
	2	1.9719	1.9719	1.9722	1.9837	1.9529	1.9259	1.771	1.991	1.647
	3	1.979	1.9854	1.8712	1.9165	1.9849	1.9641	1.928	1.9421	1.9238
	4	1.8551	1.8492	1.7968	1.8379	1.8432	1.8322	1.8483	1.8325	1.8573
MR-T1–MRA	5	1.828	1.7857	1.8276	1.7975	1.8266	1.8319	1.815	1.7928	1.8358
MRI–CT	6	1.5796	1.5913	1.6012	1.6135	1.6028	1.6103	1.6156	1.6035	1.6172
	7	1.7205	1.7257	1.7554	1.7635	1.7334	1.7877	1.7891	1.7765	1.7898
MRI–PET	8	1.8301	1.469	1.8652	1.7963	1.8568	1.8373	1.8703	1.8407	1.8658
	9	1.746	1.7579	1.7582	1.8274	1.9076	1.8856	1.9068	1.9349	1.8943
	10	1.7074	1.7367	1.7477	1.7852	1.8616	1.8304	1.8514	1.8564	1.8659
	11	1.6882	1.7435	1.7382	1.8276	1.9103	1.8464	1.883	1.8975	1.8897
MR-T2–SPECT	12	1.7056	1.7132	1.382	1.8724	1.8928	1.8931	1.9341	1.713	1.9416
	13	1.866	1.8576	1.4825	1.7948	1.8062	1.8309	1.8924	1.8869	1.8973
	14	1.814	1.8224	1.8123	1.8375	1.9058	1.8569	1.8891	1.8612	1.8924
	15	1.3545	1.7703	1.8891	1.8627	1.809	1.8819	1.8735	1.7543	1.935
	16	1.5295	1.6545	1.5409	1.6273	1.5826	1.6414	1.6217	1.6147	1.6559
**Average Value**	-	1.7582	1.7748	1.7495	1.8168	1.8393	1.8381	1.8406	1.8282	**1.8421**

**Table 10:** Performance evaluation of the fusion methods in the ranking strategy.

	Fusion Techniques								
Performance Measures	PCA	DWTPCA	DWT + Fuzzy	CONT	Chaira’s IFS	Bala’s IFS	Sugeno’s IFS	PC-NSCT	Proposed Method
API	6	9	7	4	5	3	2	8	**1**
SD	8	9	7	5	4	3	2	6	**1**
AG	7	9	6	4	8	5	2	3	**1**
SF	7	9	6	5	8	4	2	3	**1**
MSF	8	9	6	5	7	4	2	3	**1**
CC	5	8	9	7	6	3	4	2	**1**
MI	5	7	9	6	8	3	2	4	**1**
FS	8	7	9	6	3	4	2	5	**1**

**Table 11:** Average running time (seconds) of the proposed method with different existing methods.

Medical Image Modality	Fusion Techniques								
	PCA	DWTPCA	DWT + Fuzzy	CONT	Chaira’s IFS	Bala’s IFS	Sugeno’s IFS	PC-NSCT	Proposed Method
Average Value	0.80	0.60	1.48	17.69	0.87	0.65	0.50	36.72	1.19

## Data Availability

Not applicable.
